# Perceptions of Oncology Providers and Cancer Survivors on the Role of Nutrition in Cancer Care and Their Views on the “NutriCare” Program

**DOI:** 10.3390/nu12051277

**Published:** 2020-04-30

**Authors:** Laura Keaver, Ioanna Yiannakou, Sara C. Folta, Fang Fang Zhang

**Affiliations:** 1 Department of Health and Nutritional Science, Institute of Technology Sligo, Sligo, F91 YW50, Ireland; 2 Friedman School of Nutrition Science and Policy, Tufts University, Boston, MA 02111, USA; Sara.Folta@tufts.edu (S.C.F.); fang_fang.zhang@tufts.edu (F.F.Z.); 3 Department of Medicine, Boston University, Boston, MA 02215, USA; ioannay@bu.edu

**Keywords:** nutrition, oncology, cancer, intervention

## Abstract

Cancer patients and survivors can experience treatment related side effects that impact nutrition status, as well as unwanted weight loss, weight gain and poor dietary quality. Therefore, they are a group that would benefit from nutrition intervention. A qualitative study was conducted online (six focus groups and two interviews) with 12 oncology providers and 12 survivors in the United States. Participants were asked about the role of nutrition in survivors’ health, appropriate components of nutrition care for cancer patients, and strategies to integrate nutrition into oncology care. Feedback on a proposed program, “NutriCare”, was also sought. Focus groups were recorded, transcribed verbatim, and analyzed for themes. Four main themes emerged: (1) nutrition is an important component of oncology care and source of empowerment for cancer patients; (2) in the NutriCare program, the prescription pad component was viewed as a critical aspect, and there was also a preference for dealing with patients and survivors separately; (3) for implementation, the most appropriate time for providers to talk to patients about nutrition is during the development of the treatment plan. Reinforcement of key nutrition messages by providers was also highlighted; (4) major barriers included lack of time and motivation by providers. Survivors were interested in and providers supportive of integrating nutrition into oncology care.

## 1. Introduction

Cancer patients can experience side effects of treatment that can impact nutritional intake [1,2], as well as unwanted weight loss due to cachexia [3]. In addition, survivors have been shown to experience unwanted weight gain [4] and to have poorer dietary quality than the general population [5]. Nutrition therefore should form an integral part of their management and treatment plan, but this is not always the case. Previous work has found that medical oncologists and oncology teams often neglect to include either screening or information provision related to nutrition in assessments [6,7].

This lack of guidance can lead to nutrition information being obtained elsewhere, often from unreputable sources, or patients may turn to alternative approaches such as supplement use and fad diets, often unsupported by scientific evidence [8]. Previous work with cancer survivors has indicated that they are interested in receiving evidence-based nutrition information [9] and that they want to be given this information by their oncologist or oncology team [9]. This is not currently a standard part of clinical or healthcare practice in many locations.

To address this gap, the research team developed the NutriCare program using the 5A model (“ask, advise, assess, assist, arrange”), as a potential program to help providers integrate nutrition into routine oncology care using a patient-centered approach. This paper reports on focus groups and interviews conducted with oncology providers and cancer survivors to gain insight into their views on integrating nutrition into oncology care, as well as their feedback on the content and implementation of the NutriCare program.

## 2. Materials and Methods 

### 2.1. NutriCare Program

#### 2.1.1. Development of Nutrition Intervention

A nutrition intervention, “NutriCare”, was designed by the multidisciplinary team and utilized in the focus groups to determine acceptability of such an approach. The 5A model recommended by the Centers for Disease Control and Prevention for smoking cessation, with demonstrated feasibility in primary care settings [10], was adapted for use in the oncology care setting for nutrition counseling in this study. The proposed content was designed to be useful for individuals at all stages of the cancer journey, from newly diagnosed to long-term survivors, with the healthcare team indicating the appropriate sections to the survivor during the consultation process.

##### NutriCare

The NutriCare program content was developed by the research team between March and July 2018. The model is outlined below, along with the resources that have been developed to support this program.

#### 2.1.2. Ask

Patients complete a Nutrition Assessment for Cancer Patients (NACP) form that asks patients how often they experience 12 cancer-related symptoms that affect nutritional intake (e.g., nausea, vomiting, diarrhea, fatigue, or pain) and assesses adherence to the recommended intake of 11 key food groups on a 4 point Likert scale. Symptoms were chosen from nutrition impact symptoms commonly reported by cancer patients [11,12]. Questions on food groups were adapted from the validated Rapid Eating Assessments for Patients (REAP) questionnaire [13].

#### 2.1.3. Advise

**Figure 1:**
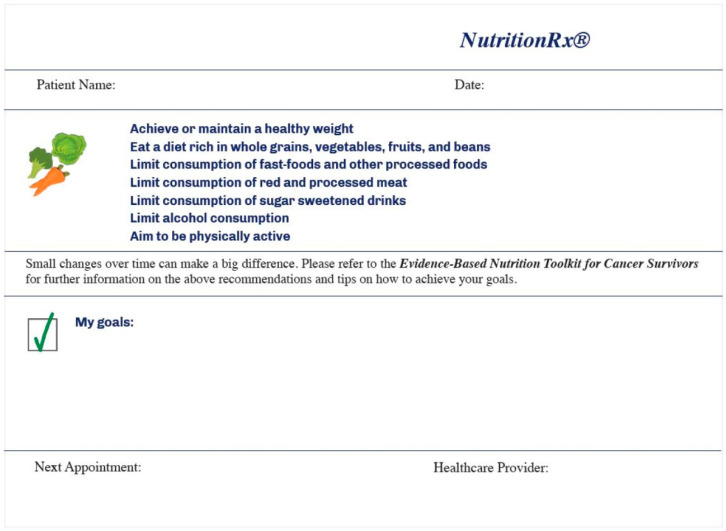
Nutrition prescription pad developed as part of the NutriCare program.

Prescription pads have been previously used to provide physical activity advice. The nutrition prescription contains seven nutrition recommendations, adapted from the current nutrition recommendations for cancer survivors and the newly released Cancer Prevention Recommendations by the World Cancer Research Fund (WCRF)/AICR with a strong evidence base [14], including (1) achieve or maintain a healthy weight; (2) eat a diet rich in whole grains, vegetables, fruits, and beans; (3) limit consumption of fast-foods and other processed foods; (4) limit consumption of red and processed meat; (5) limit consumption of sugar-sweetened beverages, (6) limit alcohol consumption; and (7) aim to be physically active (Figure 1).

In addition, the patient also receives individual-specific advice based on the results of the NACP questionnaire above. For example, if the individual highlights that nutrition impact symptoms are an issue, then they are provided with nutrition advice in this area [15,16], which is added to the prescription pad. They are also directed to the appropriate section in the patient toolkit, which is based on evidence-based practice (see Section 2.1.5 below), and the clinician then discusses how to set specific measurable, attainable, relevant, and time-based (SMART) goals around this (see Section 2.1.4 below).

#### 2.1.4. Assess

**Figure 2:**
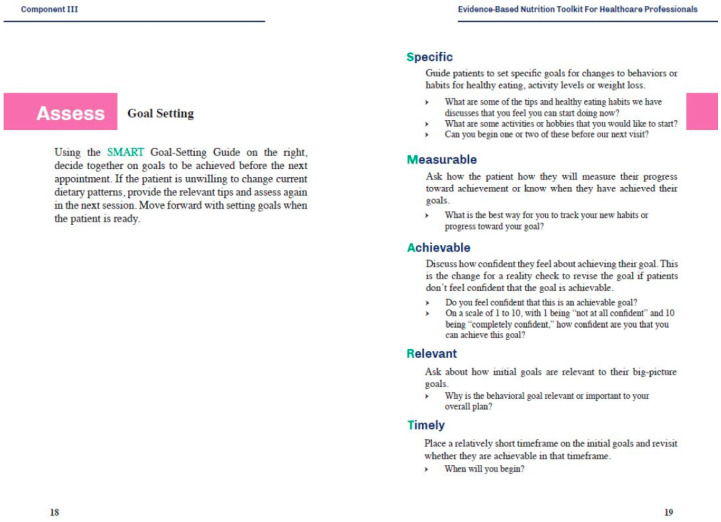
Goal-setting section of the healthcare professional toolkit.

A section on goal setting is included in the provider toolkit (Figure 2) to help with this process, and a question about willingness to change is asked in the NACP. If the individual indicates in the NACP that they are not willing to make changes, then they are still provided with the patient toolkit, but no goals are set at that time-point. If they are willing to change, then SMART goals in areas that were highlighted as important in the NACP are chosen.

#### 2.1.5. Assist

This stage involves the provision of an evidence-based patient toolkit. The toolkit comprises sections on the importance of nutrition for cancer patients and survivors, how cancer treatment can impact on eating patterns, strategies for managing these eating problems if experienced, preventing unwanted weight loss and weight gain, healthy eating and active living post-treatment, and a frequently asked question (FAQ) section split into 15 food groups or related topics, as well as additional information on food safety, how to talk to their healthcare team about their diet, and how to evaluate nutrition information for cancer survivors.

#### 2.1.6. Arrange

Finally, the clinician arranges follow-up visits to reassess the individual’s intake and refill the nutrition prescription pad.

### 2.2. Focus Groups

To help determine current views on integrating nutrition into oncology care and to obtain feedback on the NutriCare program, a total of six focus groups (three with providers and three with survivors) as well as two structured interviews (with individuals who were unable to attend the focus groups) were conducted. Convenience sampling was used, as the individuals had previously served as part of a stakeholder panel for the Nutritional Needs of Cancer Survivor Project (Project NNOCS) run by the Principle Investigator (PI). Both healthcare providers and cancer survivors were recruited in June and July 2018 and informed consent was obtained before focus groups were conducted online using WebEx between 12 July and 1 August 2018.

All focus groups lasted an hour and were moderated by the lead author (LK), who was trained by an expert in qualitative methodology (SCF). Two separate moderator guides (one for providers and one for survivors) were developed by the lead author and PI with input from the rest of the team. The providers received the healthcare professional toolkit and the 5A model guide to review prior to the focus group, while the survivors received the patient toolkit for review.

Providers were asked about their general views on nutrition practice in oncology care, including their current practice and access to evidence-based recommendations, the program content and format, and the feasibility of utilizing the toolkit in practice. Survivors were asked about the nutritional needs of someone who has survived cancer, where information is currently obtained, the content of the patient toolkit, and their thoughts on participating in the NutriCare program. At the end of the focus group, the moderator summarized the main discussion points and asked for feedback to ensure that the participants’ perceptions of key points were captured correctly. All participants received a $25 gift card for their participation.

### 2.3. Analysis

The focus groups were recorded and transcribed verbatim. Based on the research aims and the questions asked during the focus groups, a codebook was developed by the lead author, who served as the lead analyst. The codebook was inductively refined based on the focus groups and consisted of three main categories: role of nutrition in oncology care, program content, and program implementation. All research team members reviewed and approved the final codebook. A subset of the transcripts (*n =* 2) were then analyzed using this codebook by the lead analyst and one other member of the study team (IY), both of whom independently reviewed the transcripts and coded the data before meeting to obtain consensus. Any area of disagreement was resolved by reviewing the transcripts. As >80% consensus was agreed, the lead analyst coded the remaining transcripts. Microsoft Excel (version 1908) was used to assist with coding, since the number of groups and interviews was small enough not to require specialized qualitative analysis software. The coded data were then assessed to identify themes relevant to the research question. Supporting quotes for these themes were identified. Themes from both the healthcare professional and the survivor focus groups were similar and were grouped together into four categories: (1) nutrition in oncology care; (2) NutriCare program content; (3) NutriCare program implementation, and (4) barriers and facilitators. These are presented with supporting quotes. Participants’ identities were protected using participant numbers.

### 2.4. Ethics

This project was approved the Institutional Review Board of Tufts University (Institutional Review Board Protocol No. 13001).

## 3. Results

Twelve oncology providers and 12 cancer survivors (survivors of a variety of cancers of varying duration) took part in focus groups, which ranged in size from three to five participants. Two cancer survivors took part in individual interviews due to an inability to take part in the focus groups on the assigned date. Participant characteristics are outlined in Table 1.

### 3.1. Nutrition in Oncology Care

Three themes were identified in this category: (1) access to evidence-based recommendations and advice, (2) variability in nutrition provision, and (3) empowerment and control.

Both providers and patients reported challenges in accessing evidence-based nutrition recommendations. Both the oncology providers and cancer survivors considered that accessing evidence-based nutrition recommendations was important, and yet accessing these recommendations was a challenge for both, with the exception of dietitians.

*“I think there’s pretty decent access for me, as a dietitian, am because I know where to look and I know where to go, I know the organizations I like to rely upon, the physicians on the other hand, they probably don’t have the same knowledge of where to go, and establish their go to places to look up information”*

Healthcare Professional 1—dietitian

Survivors seemed to be vulnerable to misinformation and access to evidence-based guidance was not always readily available. Many reported using the internet to locate this information, particularly when it wasn’t addressed in the clinical setting, and that led them to information that was not necessarily legitimate.

*“I do know a lot of people aren’t very discerning about how they get their nutrition information, if it’s in writing and in particular if it’s on an infographic they’ll take it as gospel so you know that’s real problem”*

Survivor 1

Oncology clinics varied in providing nutrition guidance to cancer survivors. There was variability in accessing nutrition advice through oncology clinics due to different structures, resources, and locations. Some clinics had healthcare professionals specifically assigned to address behavior change or screen all patients for malnutrition or nutritional deficiencies as a standard part of practice; however, others did not address nutrition nor did they have the resources to provide such information. Some survivors had received dietitian consultations at some point during their care, but many had not.

*“it doesn’t get much attention from the medical profession at least the people that I dealt with”*

Survivor 12

Lack of nutrition input was particularly noted by healthcare professionals in rural hospitals with smaller teams, although those in larger hospitals also noted a lack of information. Even where the resources were available, they were not always utilized by healthcare professionals. In some cases, they were also not utilized by patients due to the large number of appointments they already had in addition to chemotherapy, on top of which adding a nutrition appointment did not seem feasible.

*“With the large cancer hospital that I’ve been associated with, they don’t emphasize it, and then with that people are left on their own and you know it’s not emphasized there is so much going on in cancer experience that it often left to the wayside and not dealt with”*

Survivor 3

Cancer survivors considered nutrition to be a tool for empowerment and control. Cancer survivors considered nutrition to be a tool that could empower them and give them control over an aspect of their care at a time when decisions seemed to be made by others (the oncology team). The nutrition aspect of their care provided a sense that they were taking back control of their bodies again.

*“Patients are looking for something that they can help themselves with that’s not one more chemical that they’re putting in their body, how else can I help myself a different way.”*

Survivor 9

*“It is the one aspect that they have control over and they are in charge of so I think that gives them some incentive to follow through because we can’t do it for them, they’re doing it for themselves.”*

Healthcare Professional 7—dietitian

### 3.2. NutriCare Program Content

There were two emerging themes in this area: (1) the prescription pad component was viewed as a critical aspect of the program and (2) an awareness that the needs of patients and survivors are different and should potentially be more clearly divided in this program.

The prescription pad was viewed as a critical aspect of the program. The prescription pad seemed to carry a lot of weight and there was a perception that if a clinician were to provide advice in this form it would be more likely to be taken seriously and to be implemented. It was viewed as more than just a piece of paper. It was clear that clinicians have a lot of power in this setting and that recommendations from them would be trusted, with all survivors wanting to hear this from their clinicians even ahead of nutrition experts such as dietitians.

*“I like this nutrition prescription pad, I’ve had good luck when you get a survivor to commit to a health goal and you sort of write a contract about it and this is essentially giving something and you’re going to have a discussion about specific goals, so I think that this can be very impactful.”*

Healthcare Professional 4—oncologist

*“If a healthcare provider writes a nutrition prescription, it’s hard to ignore, you know somebody else from your circle can assess the intake and weight status but the oncologist writes a nutrition prescription that’s something you have in hand that you access, so it would have some weight to it I believe.”*

Survivor 3

In regards to the differing needs of cancer patients and survivors, many of the healthcare professionals and survivors showed a preference for splitting the resource into two separate tool kits—one for those undergoing active treatment and one for those who have completed active treatment and who could focus on dietary quality and weight management. However, some still liked to have all the materials in one place and likened it to a manual for a car, where one can go to the relevant sections but still have a comprehensive resource. Some of the questions in the NACP, such as how many portions of fruit they consume per day may not be as relevant during active treatment and is not something that would specifically be addressed at that point. Therefore, further refinement of the questionnaire to reflect the differences between patients and survivors was recommended.

*“I like the fact that it sets up to be both symptom management and survivorship.”*

Survivor 7

### 3.3. NutriCare Program Implementation

Four main themes emerged that focused on implementation of the program: (1) the role of the HCP should be to champion the program; (2) a need for flexible training and guidance regarding the program content; (3) the best time to implement was deemed to be post-diagnosis, in line with the development of the treatment plan; and (4) reinforcement of key messages and the benefit of small changes should be encouraged throughout.

The healthcare professional’s role should be to champion the program. All participants felt that to be adopted, doctors would need to initiate and champion the program. Dietitians highlighted the potential impact of this, noting that consultations went better with individuals who had received a few minutes talk from their doctors highlighting the importance of nutrition before a referral to them. After a referral to the program, a dietitian or nurse could then deliver the additional components, and this could be dictated by the clinic structure and resources.

*“I think it’s fantastic and I think doctors they still have a lot of power, a lot of respect and a lot of power, especially when someone tells you you have cancer and I think they are the most important piece of the puzzle, I think if doctors start this process…”*

Survivor 1

Flexible training and guidance will be required. A need for training was recognized by the healthcare professionals who felt that this would enable anyone to deliver the intervention. It would also address the lack of confidence that many of the non-dietitians felt in their ability to deliver a nutrition intervention, when many had not received specific nutrition training and were not confident they could deal with follow-up questions. They preferred a brief, on-line training format that would fit into busy clinic schedules, with units that could be referred to when needed. Not all healthcare providers were confident in delivering behavior change interventions or developing SMART goals, and they wanted these to be incorporated in the training as well. Dietitians also felt that training would be useful, as they did not always have specific training in oncology or certain aspects of oncology, e.g., survivorship versus acute care. Survivors highlighted a need to receive guidance on how to best use the survivor toolkit and determined this should take place in person with a healthcare professional, an aspect already part of the NutriCare program.

*“I think training would be very beneficial because we can read and we can absorb but you know the fact that people would maybe ask the question I wasn’t prepared to answer would be I think training would be beneficial…”*

Healthcare Professional 6—dietitian

The best time to implement the program is alongside the treatment plan. Participants reported that patients at diagnosis are dealing with a lot and that this would not be an appropriate time to start the program, although some survivors did mention initiation at this point. The majority felt that introducing the idea of nutrition briefly at this point would be more effective, and then when the treatment plan is decided, the nutrition program should run in tandem with it and it should continue as part of the survivorship plan for it to be taken seriously and to work.

*“I think it’s a waste of time to try to discuss something like this during diagnosis because nobody will hear what you say and over my head I would not care and it would be waste of time. I think during treatment is the time to start approaching these issues”*

Survivor 12

*“Then it should just be ongoing, there should be no end to the nutrition part, it’s not something that’s temporary”*

Survivor 1

Reinforcement of messages and the benefit of small changes should be encouraged throughout. Reinforcement of the key nutrition messages emerged as a strong theme, with the aim of making the behaviors ingrained and habitual. Participants felt that it was important to have the messages continually reinforced in multiple ways by their oncology team. Patients would also need to continue asking for nutrition advice, care, and guidance.

*“[The] message has to be repeated many times especially in a cancer situation where sometimes you only take in 10 or 15 percent of the information in an appointment, so I like the toolkit. But we have to somehow get it repeated whether its oncologist, nurse practitioner, dietitian, support group, care givers, it has to be out here, you know, use your seatbelt, or don’t drink and drive, how many times have you to hear the message before it becomes automatic and that’s key”*

Survivor 3

The idea of small steps to behavior change was also very strong, especially in the face of the physical and mental challenges that oncology patients face. Participants believed that the benefit of small steps should be made very clear to the patient/survivor.

*“And maybe just an overriding sense that this whole process of changing your lifestyle habits and changing what you eat is really difficult it’s not going to be easy, and so no one get a sense of you know ‘why can’t I do this?’ It’s so incredibly hard, because it is.”*

Survivor 10

### 3.4. Barriers and Facilitators

Barriers such as time and language and facilitators, namely interest and infrastructure, were mentioned when the participants considered implementation of the NutriCare program.

The main barrier to implementing this program was time constraints. It was recognized by survivors and healthcare professionals that clinicians did not have time in busy clinics to deliver this program, and that they would not be motivated as they would not be reimbursed for their time.

*“I don’t think this will happen in practice because the physician is too busy and is not paid for a nutrition visit”*

Healthcare Professional 1—dietitian

The hospitals included in the focus groups serve a multicultural cohort and require all materials in a variety of languages. Since the toolkit is available only in English, this could serve as a barrier in some clinics.

All participants however, recognized that nutrition is an important part of oncology care in terms of quality of life and survival, and something that they would like to have incorporated into oncology practice.

*“It seems to fit hand in glove with survivorship. It’s like, in sports you try to do everything you can to prepare for an event. Seems like to me, you try to do everything you can to prepare for survival.”*

Survivor 3

Overall, there was a great interest in the NutriCare program and a consensus that it not just educated patients and survivors, but also the healthcare professionals themselves. The usability of it and the concise summary of current evidence and practical tips were lauded by both groups. In situations where there is no access to dietitians or nutrition advice, it was deemed to be beneficial to provide the toolkit to ensure that nutrition was addressed in some way.

*“I think you guys have done great work and everything, you’ve summarised a lot of really good information and it’s an excellent resource.”*

Healthcare Professional 1—dietitian

*“There are things in there that… had not occurred to me. I learned a lot from reading the toolkit and I thought it was really interesting”*

Healthcare Professional 8—nurse

There were some clinics that felt that this program would fit well with their current practice and that they had the skills and experience to deliver it and that the resources would be of benefit to this. However, there was an acknowledgement that this would not be the case for all clinics and healthcare teams.

*“It would outline very nicely with what we are already doing, I don’t think it will override some of our comfort zone to incorporate and I was looking for the resources to help and support healthy living recommendations in a way to motivate patients”*

Healthcare Professional 11—dietitian

*“I think in our institution we actually I sent this idea around to a variety of providers and everybody seemed had excited about it; the big thing is to get enough resources to spread them out throughout the cancer center where patients are seen.”*

Healthcare Professional 12—nurse

## 4. Discussion

This paper outlines the views of both oncology providers and cancer survivors on the integrating of nutrition into oncology care and their views on the use of the 5A model to deliver this. Focus group findings revealed a need and a desire for nutrition information from a reliable source from the survivors, and an interest in and support for the program from both providers and survivors. The most appropriate and feasible time to introduce this program seems to be at the point of development of the treatment plan. The content was viewed favorably, although providers suggested there might be barriers related to time and language. The appropriate role of the oncologist was indicated as that of a “champion” for the program.

All survivors indicated a desire for evidence-based information at all points in their journey. Current practices related to the provision of nutrition information are varied, and there is often limited, if any, access to nutritional counseling. Cancer survivors often reported receiving little guidance in this area and expressed an interest in receiving this information from their oncologist or physician where possible [8]. Previous literature indicates that HCPs in a range of clinical settings lack confidence and knowledge in nutrition [17,18,19]. This program aims to address this gap by providing to clinicians easy-to-use, evidence-based nutrition information and a step-by-step process to follow to aid this process. The HCP toolkit also specifies advanced nutrition issues that should be referred to a dietitian.

Nutrition is critical to the management of cancer. The detrimental effects of some treatments on dietary intake through commonly occurring side effects [2,20], as well as the cachectic response [3], can have a negative impact on patients’ nutritional status and outcomes. Maintaining muscle mass is of utmost importance, since reduced muscle mass in the form of sarcopenia is associated with fatigue, impaired physical function, reduced tolerance to treatments, impaired quality of life, and reduced survival [21]. It is important to deliver nutrition information to all rather than waiting until a patient requests it or until they have experienced substantial weight loss. In some cases, muscle wasting is not evident, for example in those who have obesity [21]. Recent work has shown that cancer survivors who receive nutritional information more often change their dietary behavior, regardless of whether they had nutritional information needs [22]. Survivors, when compared to the general population, have a diet of poorer quality [5], and they can experience weight gain from early in treatment into survivorship [4]. As cancer survivors are already at an increased risk of additional cardiovascular disease risk factors such as hypertension and type 2 diabetes [23], it is important that this is addressed to ensure the best outcomes in terms of recurrence and development of additional conditions.

Nutrition is important from a survivor perspective as it offers survivors control and it is something they can manage, and also gives a sense of empowerment. This was a very clear finding from the qualitative component of this work. By including nutrition as a part of a standard care, survivors may gain a sense of power over a component of their care. This could help to increase the motivation and empowerment of patients and survivors. Motivation plays a large role in the likelihood of an individual creating positive change in their lifestyle and the likelihood of their maintaining this change [24]. Cancer also provides a “teachable” moment when individuals are more open to change, which could have long-term health benefits [25]. While survivors reported a need for nutrition in all aspects of their care, they felt that the point of diagnosis or soon after would be the wrong time. Thus, to ensure the best outcomes, introducing the program at a time when their treatment plan is being discussed was deemed most appropriate.

The use of a prescription pad to provide nutrition advice was thought to have potential as an effective way to promote nutrition goals and for providers to be able to champion nutrition as an important aspect of care. Lifestyle prescriptions have previously shown to be effective [26], most notably in the area of physical activity promotion [27,28]. As mentioned previously, clinicians have previously reported a lack of confidence in the provision of nutrition information [17,29]; however, by utilizing an intervention where their role is to champion an intervention and to endorse a nutrition prescription, this barrier could be avoided. There could also be a place for training programs to overcome this lack of confidence.

The goal of integrating the NutriCare program into current care is to be sustainable. However, it was clear from the results that there may be barriers to this, e.g., time and language. Language could be addressed by providing the resource in additional languages other than English once the pilot work is complete and the program is refined. This program was designed to be implemented by the oncology team; however, it was clear from this work that clinicians see their role as supporting and introducing the program rather than delivering it. The potential for other team members to deliver it varies, with some clinics reporting more of a capacity to do this than others. Any intervention needs to be convenient, fit for purpose, cost-effective, and sustainable, and so pilot work will now evaluate the feasibility of incorporating this program as part of routine care and assess participation, adherence, and retention rates.

These findings will help to further inform the development of this program, with the ultimate goal of ensuring that evidenced-based nutrition information is easily accessible to individuals at all stages of their cancer journey and can be implemented in a way that suits both the patient and the medical team. Nutrition is a missing piece in healthcare—doctors prescribe medicines but not nutrition or nutritious foods [30]. A lot is yet to be done to add more nutrition and biochemistry of nutrition into the formative years of physician and healthcare professional training.

### Strengths and Limitations

The main limitation to this work is that the focus groups were conducted with a convenience sample. However, this sample represented the main professions that deal with oncology survivors and that we wanted to include. In addition, they were located across the United States.

## 5. Conclusions

This study found that survivors were interested in and providers supportive of a nutrition program for cancer survivors. They provided valuable input on the content and implementation of a proposed intervention utilizing the 5A model in a clinical setting. Determining if it is feasible to integrate nutrition care into standard oncology practice is a vital next step, and it is clear that this approach needs to be convenient and sustainable. Pilot work is currently underway to address this question and to further inform the development of the NutriCare program.

## Figures and Tables

**Table 1:** Characteristics of the 12 oncology healthcare professionals and 12 cancer survivors who took part in focus groups to explore perceptions towards integrating nutrition into standard oncology care.

Healthcare Professionals	
Characteristic	Frequency
**Sex**	Female (*n =* 10) Male (*n =* 2)
**Profession**	Dietitian (*n =* 4) Oncology nurse specialist (*n =* 4) Oncologist (*n =* 4)
**Location**	Massachusetts (*n =* 4) Philadelphia (*n =* 4) Ohio (*n =* 2) Texas (*n =* 1) Pennsylvania (*n =* 1)
**Cancer survivors**	
**Characteristic**	Frequency
**Sex**	Female (*n =* 10) Male (*n =* 2)
**Location**	Massachusetts (*n =* 4) Ohio (*n =* 2) Texas (*n =* 1) North Carolina (*n =* 1) New Jersey (*n =* 1) Illinois (*n =* 1) Philadelphia (*n =* 1) Virginia (*n =* 1)

## References

[1] Friese C., Harrison J.M., Janz N.K., Jagsi R., Morrow M., Li Y., Hamilton A.S., Ward K.C., Kurian A.W., Katz S.J. (2017). Treatment-Associated Toxicities Reported by Patients with Early-Stage Invasive Breast Cancer. Cancer.

[2] Barbera L., Seow H., Howell D., Sutradhar R., Earle C., Liu Y., Stitt A., Husain A., Sussman J., Dudgeon D. (2010). Symptom Burden and Performance Status in a Population-Based Cohort of Ambulatory Cancer Patients. Cancer.

[3] Fearon K.C.H., Strasser F., Anker S.D., Bosaeus I., Bruera E., Fainsinger R.L., Jatoi A., Loprinzi C., Macdonald N., Mantovani G. (2011). Definition and Classification of Cancer Cachexia: An International Consensus. Lancet Oncol..

[4] Vance V., Hanning R.M., Mourtzakis M., McCargar L. (2011). Weight Gain in Breast Cancer Survivors: Prevalence, Pattern and Health Consequences. Obes. Rev..

[5] Zhang F.F., Liu S., John E.M., Must A., Demark-Wahnefried W. (2015). Diet Quality of Cancer Survivors and Noncancer Individuals: Results from a National Survey. Cancer.

[6] Caccialanza R., Cereda E., Pinto C., Cotogni P., Farina G., Gavazzi C., Gandini C., Nardi M., Zagonel V., Pedrazzoli P. (2016). Awareness and Consideration of Malnutrition among Oncologists: Insights from an Exploratory Survey. Nutrition.

[7] Spiro A., Baldwin C., Patterson A., Lindsay J., Andreyev H.J.N. (2006). The Views and Practice of Oncologists Towards Nutritional Support in Patients Receiving Chemotherapy. Br. J. Cancer.

[8] Rauh S., Antonuzzo A., Bossi P., Eckert R., Fallon M., Fröbe A., Gonella S., Giusti R., Lakatos G., Santini D. (2018). Nutrition in Patients with Cancer: A New Area for Medical Oncologists? A Practising Oncologist’s Interdisciplinary Position Paper. ESMO Open.

[9] Shea-Budgell M., Kostaras X., Myhill K., Hagen N. (2014). Information Needs and Sources of Information for Patients During Cancer Follow-Up. Curr. Oncol..

[10] Lawson P.J., Flocke S.A., Casucci B. (2009). Development of an Instrument to Document the 5a’s for Smoking Cessation. Am. J. Prev. Med..

[11] Coa K.I., Epstein J.B., Ettinger D., Jatoi A., McManus K., Platek M.E., Price W., Stewart M., Teknos T.N., Moskowitz B. (2015). The Impact of Cancer Treatment on the Diets and Food Preferences of Patients Receiving Outpatient Treatment. Nutr. Cancer.

[12] Gosain R., Miller K. (2013). Symptoms and Symptom Management in Long-Term Cancer Survivors. Cancer J..

[13] Gans K., Risica P.M., Wylie-Rosett J., Ross E.M., Strolla L.O., McMurray J., Eaton C.B. (2006). Development and Evaluation of the Nutrition Component of the Rapid Eating and Activity Assessment for Patients (Reap): A New Tool for Primary Care Providers. J. Nutr. Educ. Behav..

[14] World Cancer Research Fund/American Institute for Cancer Research Food, Nutrition, Physical Activity, and the Prevention of Cancer: A Global Perspective. AICR. http://www.aicr.org/assets/docs/pdf/reports/Second_Expert_Report.pdf.

[15] Arends J., Bachmann P., Baracos V., Barthelemy N., Bertz H., Bozzetti F., Fearon K., Hutterer E., Isenring E., Kaasa S. (2017). Espen Guidelines on Nutrition in Cancer Patients. Clin. Nutr..

[16] Rock C.L., Doyle C., Demark-Wahnefried W., Meyerhardt J., Courneya K.S., Schwartz A.L., Bandera E.V., Hamilton K.K., Grant B., McCullough M. (2012). Nutrition and Physical Activity Guidelines for Cancer Survivors. CA Cancer J. Clin..

[17] Keaver L.M., O’Meara C., Mukhtar M., McHugh C. (2018). Providing Nutrition Care to Patients with Chronic Disease: An Irish Teaching Hospital Healthcare Professional Study. J. Biomed. Educ..

[18] Glanz K. (1997). Review of Nutritional Attitudes and Counseling Practices of Primary Care Physicians. Am. J. Clin. Nutr..

[19] Mowe M., Bosaeus I., Rasmussen H.H., Kondrup J., Unosson M., Rothenberg E., Irtun Ø., Group The Scandinavian Nutrition (2008). Insufficient Nutritional Knowledge among Health Care Workers?. Clin. Nutr..

[20] Kubrak C., Olson K., Jha N., Jensen L., McCargar L., Seikaly H., Harris J., Scrimger R., Parliament M., Baracos V. (2009). Nutrition Impact Symptoms: Key Determinants of Reduced Dietary Intake, Weight Loss, and Reduced Functional Capacity of Patients with Head and Neck Cancer before Treatment. Head Neck.

[21] Ryan A., Power D.G., Daly L., Cushen S.J., Bhuachalla Ē.N., Prado C.M. (2016). Cancer-Associated Malnutrition, Cachexia and Sarcopenia: The Skeleton in the Hospital Closet 40 Years Later. Proc. Nutr. Soc..

[22] Van Veen M.R., Winkels R.M., Janssen S.H.M., Kampman E., Beijer S. (2018). Nutritional Information Provision to Cancer Patients and Their Relatives Can Promote Dietary Behavior Changes Independent of Nutritional Information Needs. Nutr. Cancer.

[23] Nardi I.A., Iakobishvili Z. (2018). Cardiovascular Risk in Cancer Survivors. Curr. Treat. Options Cardiovasc. Med..

[24] Dixon A. (2008). Motivation and Confidence: What Does It Take to Change Behaviour?.

[25] Demark-Wahnefried W., Aziz N.M., Rowland J.H., Pinto B.M. (2005). Riding the Crest of the Teachable Moment: Promoting Long-Term Health after the Diagnosis of Cancer. J. Clin. Oncol..

[26] Finestone H.M., Sohmer J. (2011). The Lifestyle (Lsx) Prescription: Another Use for the Pad. CMAJ Can. Med. Assoc. J..

[27] Kallings L.V., Leijon M., Hellenius M., Ståhle A. (2008). Physical Activity on Prescription in Primary Health Care: A Follow-up of Physical Activity Level and Quality of Life. Scand. J. Med. Sci. Sports.

[28] Rödjer L., Jonsdottir I.H., Börjesson M. (2016). Physical Activity on Prescription (Pap): Selfreported Physical Activity and Quality of Life in a Swedish Primary Care Population, 2-Year Follow-Up. Scand. J. Prim. Health Care.

[29] Nowson C.A., O’Connell S.L. (2015). Nutrition Knowledge, Attitudes, and Confidence of Australian General Practice Registrars. J. Biomed. Educ..

[30] Kahan S., Manson J.E. (2017). Nutrition Counseling in Clinical Practice: How Clinicians Can Do Better. JAMA.

